# Imaging near titanium total hip arthroplasty at 0.55 T compared with 3 T

**DOI:** 10.1002/mrm.30438

**Published:** 2025-03-28

**Authors:** Kübra Keskin, Sophia X. Cui, Bochao Li, Jordan S. Gross, Jay Acharya, Zorica Buser, Jay R. Lieberman, Brian A. Hargreaves, Krishna S. Nayak

**Affiliations:** ^1^ Electrical and Computer Engineering University of Southern California Los Angeles California USA; ^2^ Siemens Medical Solutions USA Los Angeles California USA; ^3^ Biomedical Engineering University of Southern California Los Angeles California USA; ^4^ Diagnostic Radiology University of California Los Angeles Los Angeles California USA; ^5^ Orthopedic Surgery, Grossman School of Medicine New York University New York New York USA; ^6^ Gerling Institute New York New York USA; ^7^ Orthopedic Surgery University of Southern California Los Angeles California USA; ^8^ Radiology Stanford University Stanford California USA

**Keywords:** 0.55 T, 3 T, imaging near orthopedic metallic implants, titanium, total hip arthroplasty

## Abstract

**Purpose:**

To compare 0.55 T and 3 T MRI for imaging patients with titanium total hip arthroplasty (THA). Patients with orthopedic metallic implants often require diagnostic imaging to evaluate adjacent tissues. MRI performance measures, including artifact levels and SNR, vary with field strength.

**Methods:**

Six patients with titanium THA were scanned with similar protocols at 0.55 T and 3 T, including proton density (PD) weighted turbo spin echo (TSE), PD TSE with view‐angle tilting (TSE + VAT), PD slice encoding for metal artifact correction (SEMAC), and short tau inversion recovery with SEMAC (STIR‐SEMAC). Images from both field strengths were scored by two readers and qualitatively and quantitatively compared.

**Results:**

Diagnostic confidence was significantly higher at 0.55 T compared to 3 T. Perceived metal artifact was substantially reduced at 0.55 T compared to 3 T. At 0.55 T, diagnostic imaging was achieved both without and with multi spectral imaging (MSI) for PD weighted images.

**Conclusion:**

Compared to 3 T, 0.55 T MRI offers substantially reduced metal artifacts and higher diagnostic confidence when imaging titanium THA. Advanced multi‐spectral techniques may not be required when the metallic components are entirely titanium.

## 1 INTRODUCTION

Orthopedic metallic implants improve quality of life and increase mobility and possibly longevity. They are commonly used to treat damage or deformity to joints, bones, or cartilage. Total joint arthroplasty (knee and hip) and spinal fusion are among the most common procedures.^1^ In the United States (US) alone, from 2012 to 2021, there were approximately 2.5 million joint replacement surgeries.^2^ However, minor and major complications can occur after surgery, with reported rates varying from 2.7% to 4.2% for total hip arthroplasty (THA),^3^ 5.6% for total knee arthroplasty (TKA),^4^ and 4.6% for spinal implants.^5^ Accurate non‐invasive imaging with sufficient soft‐tissue contrast is an essential tool for the evaluation of the cause of complications and in surgical decision‐making and planning.

MRI offers an essential non‐invasive imaging tool to provide soft tissue contrast to evaluate the tissues near these metallic implants. However, artifacts occur because of off‐resonance, which is caused by magnetic susceptibility differences between tissues and implants and is proportional to the field strength and magnetic susceptibility of the metal composition.^6^, ^7^, ^8^ These are significant at conventional MRI field strengths of 1.5 and 3 T. Multi‐spectral imaging (MSI) techniques such as slice encoding for metal artifact correction (SEMAC),^9^ multi‐acquisition variable resonance image combination (MAVRIC),^10^ and multi‐acquisition with variable resonance image combination selective (MAVRIC‐SL)^11^ are commonly used to reduce the artifacts. These MSI techniques correct artifacts by resolving the location of off‐resonance spins with extra encoding dimension and knowledge of the spectral and spatial excitation profile. A larger number of extra encoding steps are required for larger off‐resonance, which also increases scan time.

Contemporary low‐field MRI systems (<1 T), show promise based on reduced artifact.^12^ Recent work with hip implant phantoms at 0.55 T has demonstrated that metal artifacts are significantly reduced with fewer spectral encodings required for effective artifact mitigation.^13^ Residual artifacts depend on the material composition, with titanium alloys producing the smallest artifacts compared to cobalt‐chromium and stainless steel.^14^ Titanium hip implants may be imaged using optimized turbo spin echo (TSE) sequences at 0.55 T.^13^ A recent study showed that 0.55 T MRI can help diagnose periprosthetic hip joint infection (PJI) in symptomatic THA patients.^15^ Furthermore, a recent study comparing metal artifacts caused by spinal hardware at 0.55 T to higher fields (1.5 T/3 T) showed that 0.55 T could be used in clinical practice without compromising image quality or diagnostic efficacy.^16^ Another study comparing images of patients with spinal fusion at 0.55 and 1.5 T demonstrated that metal artifacts are substantially reduced, and 0.55 T provides better imaging near the multi‐level posterior fusion hardware.^17^

In this study, we imaged patients with THA implants at both 0.55 T and 3 T field strengths using similar protocols. Reader scores were used to evaluate diagnostic confidence, the severity of metal artifacts, perceived image sharpness, and perceived SNR at both field strengths. We specifically focused on patients with titanium THA because of the predominance of titanium implants used at our institution in recent years. Additionally, we assessed the necessity for MSI at 0.55 T to achieve diagnostic imaging for patients with titanium THA.

## 2 METHODS

### 2.1 Recruitment

Six patients with titanium THA were scanned at 0.55 and 3 T under a protocol approved by our institutional review board after providing written informed consent. All patients recruited were asymptomatic volunteers and within 1 year of their THA surgery. All patients had titanium alloy acetabular cup, ceramic femoral head, titanium alloy femoral stem, and titanium screw. Patients were scanned at 0.55 and 3 T on the same day back‐to‐back.

### 2.2 MRI scanners

0.55 T imaging was performed on a whole‐body 0.55 T scanner (prototype MAGNETOM Aera, Siemens Healthineers, Erlangen, Germany) equipped with shielded gradients with maximum 45 mT/m amplitude and 200 T/m/s slew rate. Data were collected using six elements of a table‐integrated spine array (posterior) and a six‐channel body coil (anterior). 3 T imaging was performed on a whole‐body 3 T scanner (MAGNETOM Prisma Fit, Siemens Healthineers, Erlangen, Germany) equipped with gradients capable of 80 mT/m amplitude and 200 T/m/s slew rate. Data were collected using four elements of a table‐integrated spine array (posterior) and an 18‐channel body coil (anterior).

### 2.3 MRI protocols

At both field strengths, we used standard clinical sequences, including TSE, TSE with view‐angle tilting (TSE + VAT), slice encoding for metal artifact correction (SEMAC), and short tau inversion recovery with SEMAC (STIR‐SEMAC). Imaging was performed with a coronal prescription with respect to the alignment of the implant stem. Detailed scan parameters are listed in Table 1. All TSE acquisitions were less than 4 min, and all MSI acquisitions were less than 10 min. Note that the STIR‐SEMAC at 0.55 T has a larger voxel size compared to 3 T. Additionally, the sequences were not time‐matched between 0.55 and 3 T.

**TABLE 1:** Scan parameters (TR, TE, inversion time, FOV, matrix size, refocusing flip angle, readout bandwidth, phase encode direction, turbo factor, acceleration, number of averages, VAT, SEMAC factor, and scan time) for in vivo experiments at 0.55 and 3 T.

Parameter∖sequence	0.55 T			3 T		
	TSE|TSE+VAT	SEMAC	STIR‐SEMAC	TSE|TSE+VAT	SEMAC	STIR‐SEMAC
TR (ms)	2000	2100	4330	4000	4000	6000
TE (ms)	34	31	31	37	40	25
Inversion time (ms)	–	–	110	–	–	220
FOV (mm)	280 × 228 × 80	280 × 228 × 80	280 × 228 × 80	280 × 228 × 80	280 × 228 × 80	280 × 228 × 80
Matrix size	320 × 260 × 20	320 × 260 × 20	192 × 156 × 20	320 × 260 × 20	320 × 260 × 20	256 × 208 × 20
Refocusing flip angle (°)	180	180	180	120	120	135
Readout bandwidth (Hz/Px)	401	401	401	710	710	592
Phase encode direction	R≫L	R≫L	R≫L	R≫L	R≫L	R≫L
Turbo factor	8	11	13	16	16	20
Acceleration	1	2	2	1	4	4
Averages	2	1	1	1	1	1
VAT	Off | On	On	On	Off | On	On	On
SEMAC factor	–	6|8	6|8	–	16	16
Scan time (min:s)	3:52	4:41|6:13	5:18|7:02	2:02	9:42	9:44

Abbreviations: SEMAC, slice encoding for metal artifact correction; STIR‐SEMAC, short tau inversion recovery with SEMAC; TSE, turbo spin echo; VAT, view‐angle tilting.

### 2.4 Reader scores

All images from the 0.55 T and 3 T scans were anonymized and randomized. All images were independently evaluated by two radiologists with 14 and 13 years of experience. Readers were blinded to field strengths and sequence parameters. The images were rated for diagnostic confidence, perceived metal artifact, perceived sharpness, and perceived SNR. All ratings were performed on a 5‐point Likert scale, where 1 being the worst, 5 being the best scores, and 1 to 2 non‐diagnostic and 3 to 5 diagnostic. Additionally, readers indicated their findings in the images if present.

### 2.5 Data analysis

The scores were analyzed by calculating the average, SD, median, and interquartile range (IQR) of all the scores from all images for each sequence applied at each field strength. Statistical differences between all the scores for 0.55 T and 3 T images for each sequence were determined via Wilcoxon signed‐rank tests with a significance level of 0.05. SNR was calculated from the thigh muscle signal and background noise. The ratio of measured SNR at 3 T over 0.55 T was calculated for TSE, TSE + VAT, and SEMAC sequences. The ratios from these sequences were aggregated to compute the average and SD of the overall SNR ratio.

## 3 RESULTS

The six patients ranged in age from 53 to 65 years old with body mass index (BMI) ranging from 24.2 to 37.1 kg/m^2^. Two of the patients were female and four were male. All patients had titanium THA material, as titanium is commonly used in the clinic from which the patients were recruited.

Table 2 displays the mean with SD and median with IQR of all reader scores from each sequence at each field strength and presents the *p*‐values calculated using Wilcoxon signed‐rank tests. Overall, all sequences for imaging of the titanium THA at 0.55 T produced images that readers rated as diagnostic based on median Likert scores (≥3 for all categories and sequences). By comparison, imaging at 3 T showed variability, with median scores of 3 for all categories except the metal artifact category across TSE, TSE + VAT, and SEMAC sequences, but lower scores in the metal artifact category except the SEMAC sequence. Specifically, median metal artifact scores at 3 T were 2 for TSE and TSE + VAT and 3 for SEMAC, suggesting that MSI is required to achieve diagnostic imaging. Additionally, all categories for all sequences yielded higher average scores at 0.55 T. Reader scores for 0.55 T images were significantly higher than 3 T images in diagnostic confidence (*p* < 0.05), perceived sharpness (*p* < 0.05), and metal artifact categories (*p* < 0.005) for all sequences. The difference in the perceived SNR category was not significant for TSE, TSE + VAT, and SEMAC sequences. Quantitatively, the mean and SD values of the ratio of measured SNR at 3 T to 0.55 T for TSE, TSE + VAT, and SEMAC were 1.8 ± 0.7, 1.9 ± 0.5, and 1.7 ± 0.7, respectively. Overall, the mean and SD of the ratio of measured SNR were 1.8 ± 0.6.

**TABLE 2:** The mean ± SD and median – IQR of two readers' scores for all images in each sequence at each field strength.

Category	Sequence	0.55 T		3 T		*p*‐value
		Mean ± SD	Median – [IQR]	Mean ± SD	Median – [IQR]	
Diagnostic confidence	TSE	4.2 ± 0.7	4.0 – [4.0, 5.0]	3.0 ± 0.6	3.0 – [3.0, 3.0]	0.004**
	TSE + VAT	4.1 ± 0.8	4.0 – [3.8, 5.0]	2.9 ± 0.5	3.0 – [3.0, 3.0]	0.012*
	SEMAC	4.2 ± 0.7	4.0 – [4.0, 5.0]	2.9 ± 0.3	3.0 – [3.0, 3.0]	0.002**
	STIR SEMAC	3.8 ± 0.4	4.0 – [4.0, 4.0]	2.5 ± 0.5	2.5 – [2.0, 3.0]	0.002**
Metal artifact	TSE	4.1 ± 0.8	4.0 – [3.8, 5.0]	2.4 ± 0.7	2.0 – [2.0, 3.0]	0.004**
	TSE + VAT	4.1 ± 0.8	4.0 – [3.8, 5.0]	2.4 ± 0.8	2.0 – [2.0, 2.3]	0.004 **
	SEMAC	4.2 ± 0.8	4.0 – [3.8, 5.0]	2.7 ± 0.5	3.0 – [2.0, 3.0]	0.002**
	STIR SEMAC	4.1 ± 0.3	4.0 – [4.0, 4.0]	2.2 ± 0.4	2.0 – [2.0, 2.0]	0.002**
Perceived sharpness	TSE	3.9 ± 0.7	4.0 – [3.8, 4.0]	3.2 ± 0.4	3.0 – [3.0, 3.0]	0.008*
	TSE + VAT	3.8 ± 0.6	4.0 – [3.8, 4.0]	3.1 ± 0.3	3.0 – [3.0, 3.0]	0.020*
	SEMAC	3.8 ± 0.6	4.0 – [3.8, 4.0]	2.9 ± 0.3	3.0 – [3.0, 3.0]	0.004**
	STIR SEMAC	3.3 ± 0.5	3.0 – [3.0, 4.0]	2.5 ± 0.5	2.5 – [2.0, 3.0]	0.031*
Perceived SNR	TSE	3.7 ± 0.9	4.0 – [3.8, 4.0]	3.3 ± 0.5	3.0 – [3.0, 3.3]	0.312
	TSE + VAT	3.4 ± 0.8	4.0 – [3.0, 4.0]	3.2 ± 0.4	3.0 – [3.0, 3.0]	0.508
	SEMAC	3.6 ± 0.8	4.0 – [3.8, 4.0]	3.0 ± 0.0	3.0 – [3.0, 3.0]	0.065
	STIR SEMAC	3.4 ± 0.5	3.0 – [3.0, 4.0]	2.6 ± 0.5	3.0 – [2.0, 3.0]	0.031*

*Note*: Categories that show statistically significant differences (*p* <0.05) based on the Wilcoxon test comparing 0.55 T to 3 T from two readers' scores for all images in each sequence are indicated with asterisks (*). Categories with *p* <0.005 are indicated with (**).

Abbreviations: IQR, interquartile range; SEMAC, slice encoding for metal artifact correction; STIR‐SEMAC, short tau inversion recovery with SEMAC; TSE, turbo spin echo; VAT, view‐angle tilting.

**FIGURE 1:**
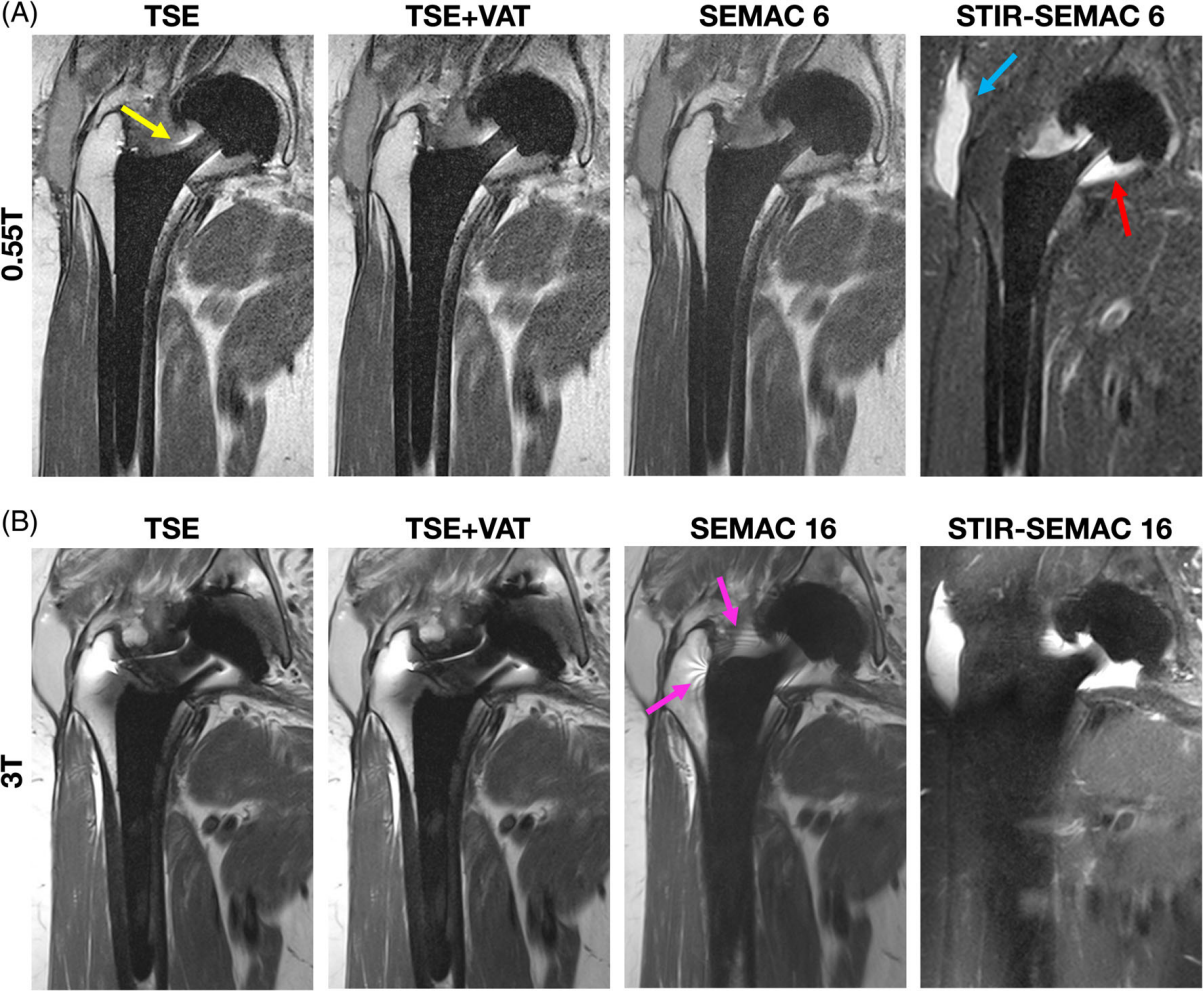
Comparison of TSE, TSE with VAT, SEMAC, and STIR‐SEMAC images at (A) 0.55 T and (B) 3 T for a 65‐year‐old male (body mass index of 30.5 kg/m^2^) with a titanium total hip arthroplasty. At 0.55 T, TSE and TSE with VAT provide results that are comparable to SEMAC. However, at 3 T, although SEMAC corrects slice encoding artifacts, it still leaves some ripple artifacts around the stem (magenta arrows), and the outline of the implant is not as clear as at 0.55 T. Note that there are small pile‐up artifacts near the neck of the implant in TSE images at 0.55 T (yellow arrow). Borders of the joint effusion (red arrow) and fluid in the trochanteric bursa (blue arrow) are sharper, and the implant head and neck junction are clearly visible at 0.55 T in STIR‐SEMAC. SEMAC, slice encoding for metal artifact correction; STIR‐SEMAC, short tau inversion recovery with SEMAC; TSE, turbo spin echo; VAT, view‐angle tilting.

Representative comparisons of TSE, TSE with VAT, SEMAC, and STIR‐SEMAC sequences at 0.55 T and 3 T for a 65‐year‐old male with a BMI of 30.5 kg/m^2^ are shown in Figure 1. Additional subjects are illustrated in Figures S1–S3. Overall, we observed a significant reduction in the severity of metal artifacts at 0.55 T compared to 3 T, with 0.55 T images rated significantly higher in the metal artifact category across all sequences (*p* < 0.005). At 0.55 T, TSE and TSE + VAT provided results comparable to SEMAC in terms of metal artifact reduction with a median Likert score of 4 for all. However, imaging at 3 T still required MSI to reduce through‐plane artifacts, and median scores for TSE and TSE + VAT in the metal artifact category were 2, compared to 3 for SEMAC. There were also remaining ripple artifacts near the stem and the femoral head at 3 T that were not observed at 0.55 T, and the outline of the implant was not as clear as at 0.55 T. Note that a small pile‐up artifact near the neck of the implant was observed with TSE at 0.55 T for all patients. However, it was not found to affect the scores in the metal artifact category. For STIR‐SEMAC images, we observed in Figure 1 that the borders of the effusion and fluid in the trochanteric bursa are sharper at 0.55 T. In Figure S3, the fluid above the implant neck is visible at 0.55 T, whereas it is obscured at 3 T.

**FIGURE 2:**
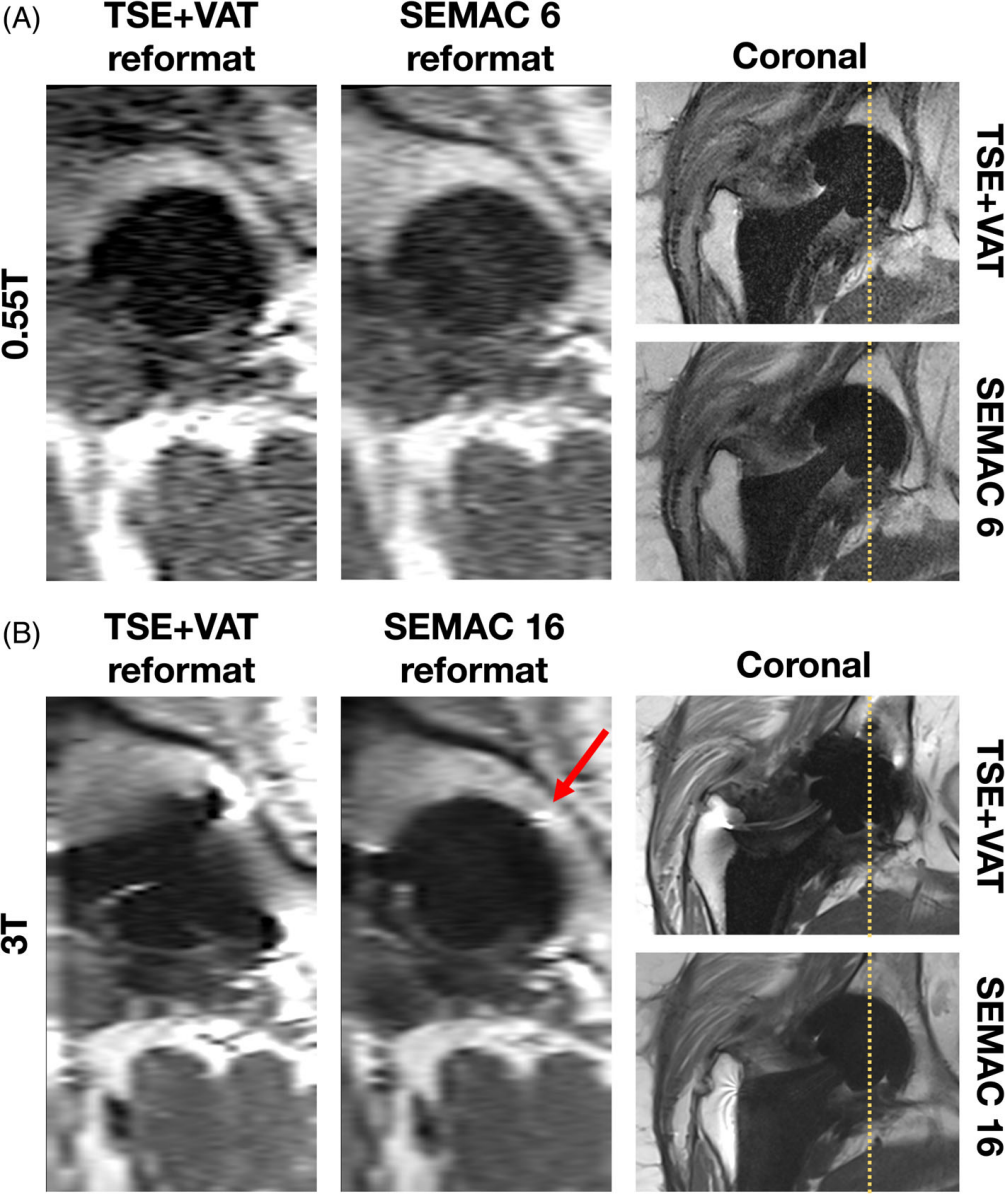
Comparison of TSE with VAT and SEMAC images at (A) 0.55 T and (B) 3 T for a 58‐year‐old female (body mass index of 31.5 kg/m^2^) with a titanium total hip arthroplasty (THA). Shown are orthogonal reformats along the slice dimension, at the locations marked by yellow dashed lines. For titanium THA at 0.55 T, both reformats look similar. However, SEMAC is needed to correct the through‐plane artifacts at 3 T. Even after SEMAC, some ripple artifacts (red arrow) remain at 3 T. SEMAC, slice encoding for metal artifact correction; STIR‐SEMAC, short tau inversion recovery with SEMAC; TSE, turbo spin echo; VAT, view‐angle tilting.

**FIGURE 3:**
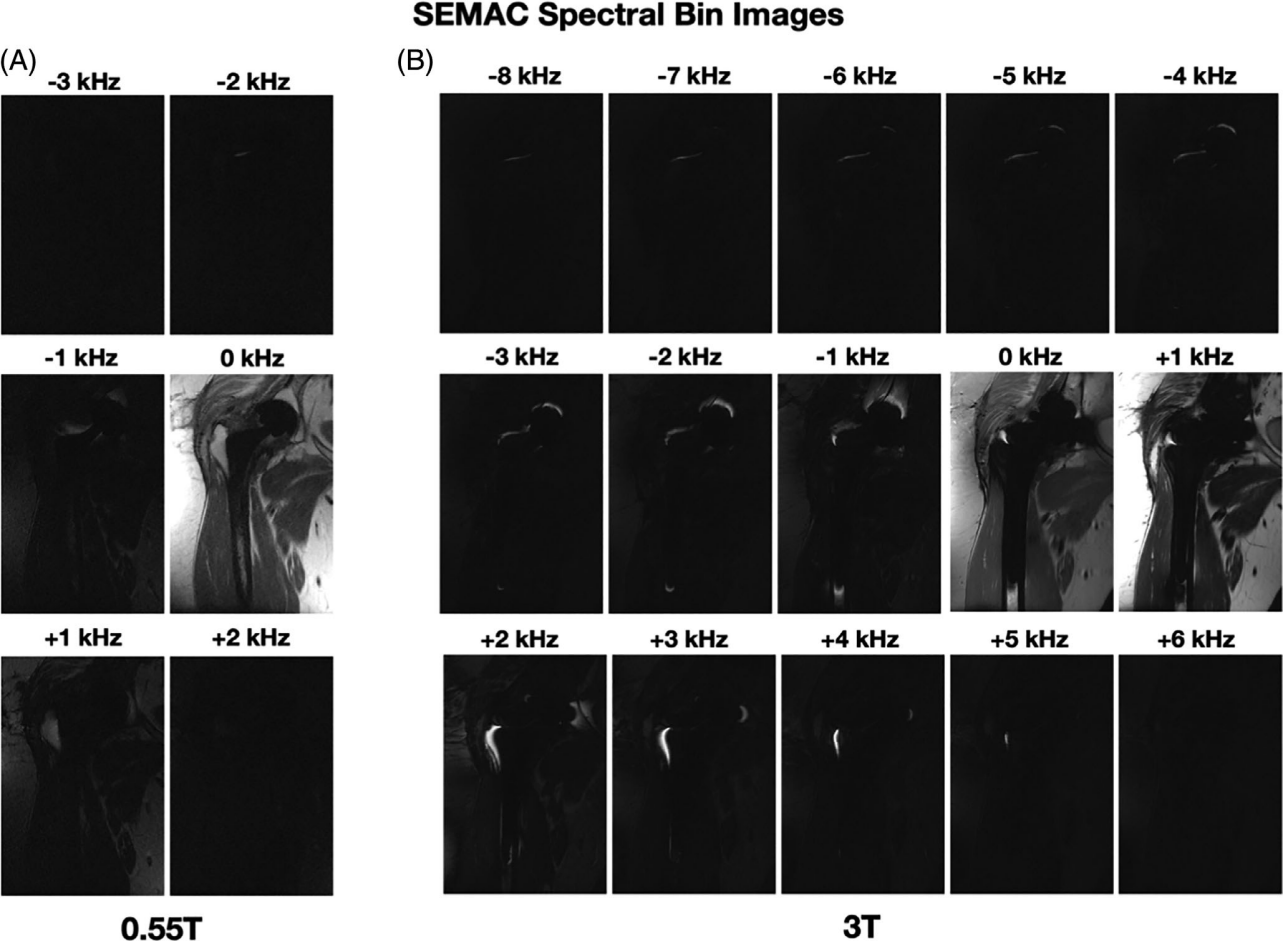
Comparison of SEMAC spectral bin images for a slice at (A) 0.55 T and (B) 3 T for a 58‐year‐old female with a titanium total hip arthroplasty. These spectral bin images were combined to obtain the final SEMAC images shown in Figure 2. Titanium implants have a low spectral range at 0.55 T, so the main contribution of signal comes from very few spectral bins. SEMAC, slice encoding for metal artifact correction.

Figure 2 illustrates images reformatted into the sagittal plane and compares TSE with VAT and SEMAC at (Figure 2A) 0.55 T and (Figure 2B) 3 T for a 58‐year‐old female with a BMI of 31.5 kg/m^2^. The location of the reformat is shown with the yellow line on the coronal images. The titanium implant has very low in‐plane and through‐plane distortions at 0.55 T, and conventional TSE provides diagnostic quality. Although through‐plane artifacts are corrected with multi‐spectral acquisition at 3 T, the remaining ripple artifacts can be observed in the 3 T SEMAC images, as shown with the red arrow. Figure 3 shows SEMAC spectral bin images for the same patient at (Figure 3A) 0.55 T and (Figure 3B) 3 T. The titanium implant has a low range of off‐resonance at 0.55 T so the main contribution of signal comes from very few spectral bins. We observed that the central spectral bin almost completely recovers the tissues very close to the implant at 0.55 T, except around the neck of the implant, which would explain the small pile‐up artifact that we observed in the TSE images at 0.55 T.

## 4 DISCUSSION

We observed a significant reduction in the severity of metal artifacts at 0.55 T compared to 3 T. The reader study suggested that 0.55 T provides better diagnostic confidence, metal artifact, and perceived image sharpness for imaging titanium hip implant patients with PD TSE, TSE + VAT, SEMAC, and STIR‐SEMAC sequences. This presents a promising advancement in imaging for patients with orthopedic metallic implants. However, there is a trade‐off between metal artifact severity and SNR, with 0.55 T exhibiting lower SNR compared to 3 T, which did not affect diagnostic quality for the tested sequences. Note that STIR imaging without MSI was not evaluated in this study, and it may suffer more from a decrease in SNR at 0.55 T. Additionally, blinded radiologists gave 0.55 T slightly higher scores for perceived SNR despite SNR at 0.55 T being measurably lower (1.8 times on average with our protocol), and the difference in perceived SNR was not statistically significant. It is possible that the readers' perceived SNR ratings reflect image quality immediately adjacent to the implant, which is their area of focus. It is also possible that the reduced metal artifacts at 0.55 T contributed to a perception of higher SNR, as images with fewer artifacts may be perceived as having higher quality.

One limitation of the study is that imaging parameters could not be perfectly matched between 0.55 T and 3 T protocols. Most of these differences are attributed to relaxation (T_1_), practical limitations (specific absorption rate), timing, and SNR. Notably, there was a large difference in voxel size for STIR‐SEMAC, primarily because of SNR and the inability to fine‐tune the spatial resolution. The larger voxel size used for STIR‐SEMAC at 0.55 T compared to 3 T might limit the visibility of fine anatomic structures while providing higher SNR.

For titanium THA at 0.55 T, simple sequences like TSE and TSE with VAT provided diagnostic quality for PD weighted images based on reader scores ≥3. This outcome is not surprising because of the relatively low magnetic susceptibility of titanium compared to other materials used in orthopedic implants. The range of off‐resonance frequencies caused by the titanium implant can mostly fall within one spectral bin. We observed that the central spectral bin almost completely recovers the tissues very close to the implant at 0.55 T, except around the neck of the implant with 1 kHz RF bandwidth, which might explain the small pile‐up artifact that we observed in the TSE images at 0.55 T. This suggests that TSE with high RF bandwidth at 0.55 T sufficiently reduces this pile‐up artifact, avoiding the need for advanced multi‐spectral imaging techniques used at conventional MRI field strengths. This might simplify the diagnostic process, reduce the duration of image acquisition, and shorten the scan protocols, leading to more efficient patient care. It might also allow other sequences to be applied without metal artifact concerns, such as quantitative relaxometry.

It is worth doing a similar study in THA with different metal compositions, such as cobalt‐chromium, which has an approximately five times greater magnetic susceptibility variation than titanium. We would expect MSI is still required as shown in a recent phantom study.^13^ It is difficult to do such an in vivo study at our institution because the vast majority of THAs use titanium, and patient recruitment for research has the highest yield in the first year after surgery.

We did not use constrained reconstruction or any form of denoising strategy. There are many promising approaches, although not in widespread use yet, that may help mitigate the reduced SNR at lower field strengths, and these could be combined with the approaches we have studied. Other studies have shown that learning‐based strategies can further improve image quality at 0.55 T.^18^, ^19^ Additionally, because the sequences were not time‐matched between two field strengths, SNR at 0.55 T could be further improved by applying more averaging, taking advantage of the time saved by requiring fewer or no spectral encoding steps.

## 5 CONCLUSIONS

In the context of hip MRI evaluation of patients with titanium THA, 0.55 T MRI provides significantly higher diagnostic confidence and significantly lower metal artifact, compared to conventional 3 T MRI. Evaluation of these patients may not require multi‐spectral imaging at 0.55 T for PD‐weighted images, which carries an associated scan time and complexity cost. The use of 0.55 T has the potential to improve radiological evaluation in these patients.

## FUNDING INFORMATION

National Institutes of Health, Grant/Award Number: R01‐AR078912; National Science Foundation, Grant/Award Number: 1828736); Siemens Healthineers; USC Annenberg Graduate Fellowship to K.K.

## CONFLICT OF INTEREST STATEMENT

S.X.C. is an employee of Siemens Medical Solutions USA.

## Supporting information


**Figure S1.** Comparison of TSE, TSE with VAT, and SEMAC images at (A) 0.55 T and (B) 3 T for a 60‐year‐old male (BMI of 24.2 kg/m^2^) with a titanium THA. At 0.55 T, TSE and TSE with VAT provide results that are comparable to SEMAC. However, at 3 T, although SEMAC corrects slice encoding artifacts, it still leaves some ripple artifacts near the stem (red arrows). The implant's outline is more clearly identifiable at 0.55 T (blue arrow).
**Figure S2.** Comparison of TSE, TSE with VAT, and SEMAC images at 0.55 T (top row) and 3 T (bottom row) for a 58‐year‐old male (BMI of 28.7 kg/m^2^) (A, B) and a 58‐year‐old female (BMI of 31.5 kg/m^2^) (C, D) with titanium THAs. At 0.55 T, TSE and TSE with VAT provide results that are comparable to SEMAC. However, at 3 T, although SEMAC corrects slice encoding artifacts, it still leaves some ripple artifacts near the stem (red arrow) and femoral head (blue arrow).
**Figure S3.** Comparison of TSE with VAT, SEMAC, and STIR‐SEMAC images at (A) 0.55 T and (B) 3 T for a 53‐year‐old female with a BMI of 37.1 kg/m^2^. The fluid collection above the implant neck (red arrow) can be seen at 0.55 T, whereas it is obscured at 3 T.

## References

[1] Overview of Operating Room Procedures During Inpatient Stays in U.S. Hospitals . Hospitals, 2018 #281. https://hcup‐us.ahrq.gov/reports/statbriefs/sb281‐Operating‐Room‐Procedures‐During‐Hospitalization‐2018.jsp Accessed May 21, 2024.

[2] Hegde V , Stambough JB , Levine BR , Springer BD . Highlights of the 2022 American Joint Replacement Registry Annual Report. Arthroplasty Today. 2023;21:101137. doi:10.1016/j.artd.2023.101137 37193538 PMC10182168

[3] O'Malley NT , Fleming FJ , Gunzler DD , Messing SP , Kates SL . Factors independently associated with complications and length of stay after hip arthroplasty: analysis of the National Surgical Quality Improvement Program. J Arthroplasty. 2012;27:1832‐1837. doi:10.1016/j.arth.2012.04.025 22810006

[4] Belmont PJ , Goodman GP , Waterman BR , Bader JO , Schoenfeld AJ . Thirty‐day postoperative complications and mortality following total knee arthroplasty: incidence and risk factors among a national sample of 15,321 patients. J Bone Joint Surg Am. 2014;96:20‐26. doi:10.2106/JBJS.M.00018 24382720

[5] Koshimizu H , Nakashima H , Ohara T , et al. Implant‐related complications after spinal fusion: a multicenter study. Glob Spine J. 2024;14:74‐81. doi:10.1177/21925682221094267 PMC10676178 35400240

[6] Koch KM , Hargreaves BA , Pauly KB , Chen W , Gold GE , King KF . Magnetic resonance imaging near metal implants. J Magn Reson Imaging. 2010;32:773‐787. doi:10.1002/jmri.22313 20882607

[7] Hargreaves BA , Worters PW , Pauly KB , Pauly JM , Koch KM , Gold GE . Metal‐induced artifacts in MRI. AJR Am J Roentgenol. 2011;197:547‐555. doi:10.2214/AJR.11.7364 21862795 PMC5562503

[8] Schenck JF . The role of magnetic susceptibility in magnetic resonance imaging: MRI magnetic compatibility of the first and second kinds. Med Phys. 1996;23:815‐850. doi:10.1118/1.597854 8798169

[9] Lu W , Pauly KB , Gold GE , Pauly JM , Hargreaves BA . SEMAC: slice encoding for metal artifact correction in MRI. Magn Reson Med. 2009;62:66‐76. doi:10.1002/mrm.21967 19267347 PMC2837371

[10] Koch KM , Lorbiecki JE , Hinks RS , King KF . A multispectral three‐dimensional acquisition technique for imaging near metal implants. Magn Reson Med. 2009;61:381‐390. doi:10.1002/mrm.21856 19165901

[11] Koch KM , Brau AC , Chen W , et al. Imaging near metal with a MAVRIC‐SEMAC hybrid. Magn Reson Med. 2011;65:71‐82. doi:10.1002/mrm.22523 20981709

[12] Campbell‐Washburn AE , Ramasawmy R , Restivo MC , et al. Opportunities in interventional and diagnostic imaging by using high‐performance low‐field‐strength MRI. Radiology. 2019;293:384‐393. doi:10.1148/radiol.2019190452 31573398 PMC6823617

[13] Khodarahmi I , Brinkmann IM , Lin DJ , et al. New‐generation low‐field magnetic resonance imaging of hip arthroplasty implants using slice encoding for metal artifact correction: first in vitro experience at 0.55 T and comparison with 1.5 T. Invest Radiol. 2022;57:517‐526. doi:10.1097/RLI.0000000000000866 35239614 PMC9363001

[14] Breit HC , Vosshenrich J , Clauss M , et al. Visual and quantitative assessment of hip implant‐related metal artifacts at low field MRI: a phantom study comparing a 0.55‐T system with 1.5‐T and 3‐T systems. Eur Radiol Exp. 2023;7:5. doi:10.1186/s41747-023-00320-5 36750494 PMC9905379

[15] Plesniar J , Breit HC , Clauss M , Donners R . Diagnosing periprosthetic hip joint infection with new‐generation 0.55T MRI. Eur J Radiol. 2024;176:111524. doi:10.1016/j.ejrad.2024.111524 38851014

[16] Kelsey LJ , Seiberlich N , Bapuraj J , Gulani V , Mishra S . Clinical Imaging of Patients with Spinal Hardware at 0.55T: Diagnostic Feasibility and Metallic Artifact Comparison to 1.5/3T. Proceedings of the ISMRM Annual Meeting. International Society for Magnetic Resonance in Medicine (ISMRM); 2024:2685.

[17] Seifert AC , Breit HC , Schlicht F , Donners R , Harder D , Jan V . Comparing metal artifact severity and ability to assess near‐metal anatomy between 0.55 T and 1.5 T MRI in patients with metallic spinal implants‐A scanner comparison study. Acad Radiol. 2024;31:2456. doi:10.1016/j.acra.2023.12.048 38242732

[18] Lopez Schmidt I , Haag N , Shahzadi I , et al. Diagnostic image quality of a low‐field (0.55T) knee MRI protocol using deep learning image reconstruction compared with a standard (1.5T) knee MRI protocol. J Clin Med. 2023;12:1916. doi:10.3390/jcm12051916 36902704 PMC10003576

[19] Schlicht F , Vosshenrich J , Donners R , et al. Advanced deep learning‐based image reconstruction in lumbar spine MRI at 0.55 T ‐ effects on image quality and acquisition time in comparison to conventional deep learning‐based reconstruction. Eur J Radiol Open. 2024;12:100567. doi:10.1016/j.ejro.2024.100567 38711678 PMC11070664

