# Supplementary material for: Imaging near titanium total hip arthroplasty at 0.55 T compared with 3 T
Source: Magn Reson Med. 2025 Mar 28;94(1):242–50. doi: 10.1002/mrm.30438 (PMC12021306; doi:10.1002/mrm.30438)
Supplement: Supplementary file 1 — Figure S1. Comparison of TSE, TSE with VAT, and SEMAC images at (A) 0.55 T and (B) 3 T for a 60‐year‐old male (BMI of 24.2 kg/m2) with a titanium THA. At 0.55 T, TSE and TSE with VAT provide results that are comparable to SEMAC. However, at 3 T, although SEMAC corrects slice encoding artifacts, it still leaves some ripple artifacts near the stem (red arrows). The implant's outline is more clearly identifiable at 0.55 T (blue arrow). Figure S2. Comparison of TSE, TSE with VAT, and SEMAC images at 0.55 T (top row) and 3 T (bottom row) for a 58‐year‐old male (BMI of 28.7 kg/m2) (A, B) and a 58‐year‐old female (BMI of 31.5 kg/m2) (C, D) with titanium THAs. At 0.55 T, TSE and TSE with VAT provide results that are comparable to SEMAC. However, at 3 T, although SEMAC corrects slice encoding artifacts, it still leaves some ripple artifacts near the stem (red arrow) and femoral head (blue arrow). Figure S3. Comparison of TSE with VAT, SEMAC, and STIR‐SEMAC images at (A) 0.55 T and (B) 3 T for a 53‐year‐old female with a BMI of 37.1 kg/m2. The fluid collection above the implant neck (red arrow) can be seen at 0.55 T, whereas it is obscured at 3 T. [file MRM-94-242-s001.docx]

**Supporting Information**


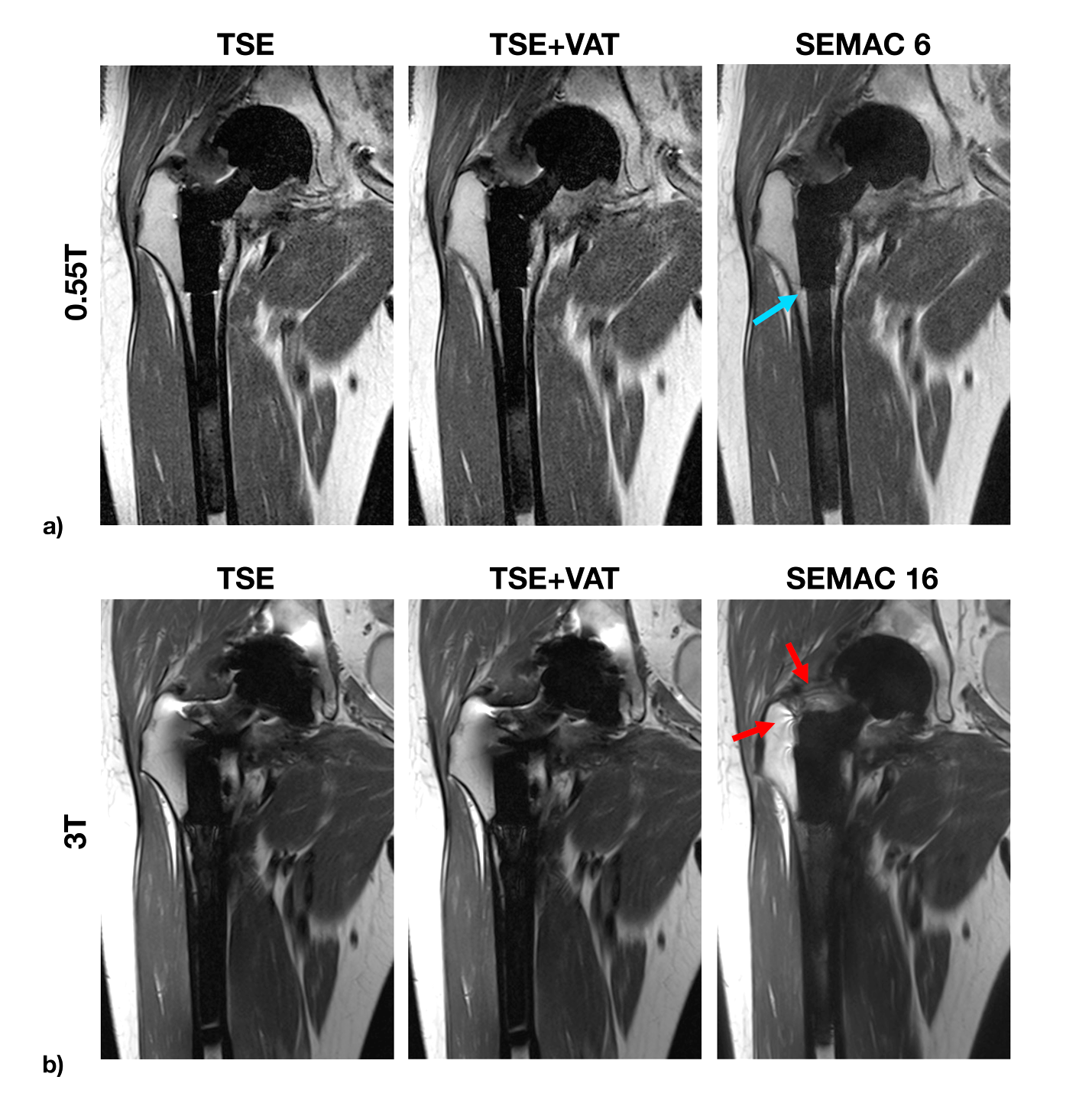


**Supporting Figure S1**: Comparison of TSE, TSE with VAT, and SEMAC images at a) 0.55T and b) 3T for a 60-year-old male (BMI of 24.2 kg/m^2^) with a titanium THA. At 0.55T, TSE and TSE with VAT provide results that are comparable to SEMAC. However, at 3T, although SEMAC corrects slice encoding artifacts, it still leaves some ripple artifacts near the stem (red arrows). The implant's outline is more clearly identifiable at 0.55T (blue arrow).


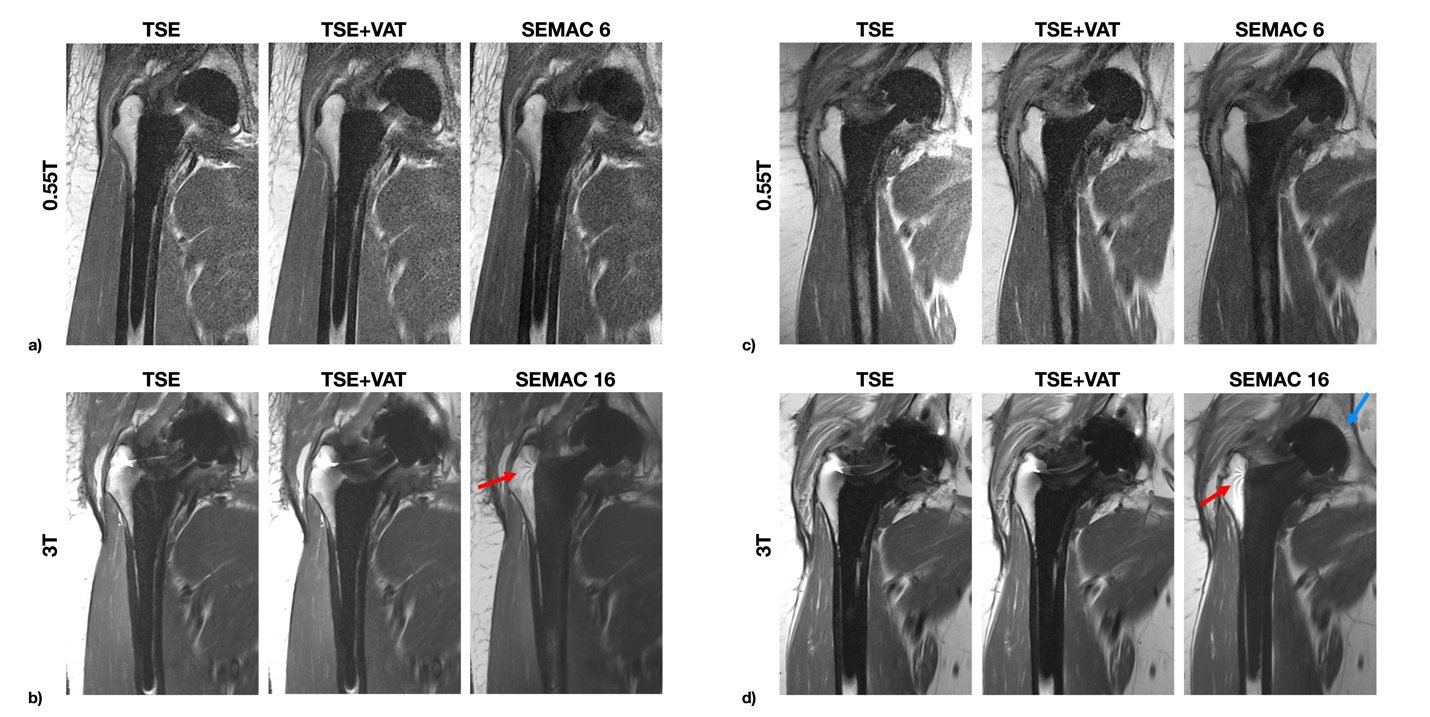


**Supporting Figure S2**: Comparison of TSE, TSE with VAT, and SEMAC images at 0.55T (top row) and 3T (bottom row) for a 58-year-old male (BMI of 28.7 kg/m^2^) (a-b) and a 58-year-old female (BMI of 31.5 kg/m^2^) (c-d) with titanium THAs. At 0.55T, TSE and TSE with VAT provide results that are comparable to SEMAC. However, at 3T, although SEMAC corrects slice encoding artifacts, it still leaves some ripple artifacts near the stem (red arrow) and femoral head (blue arrow).

**
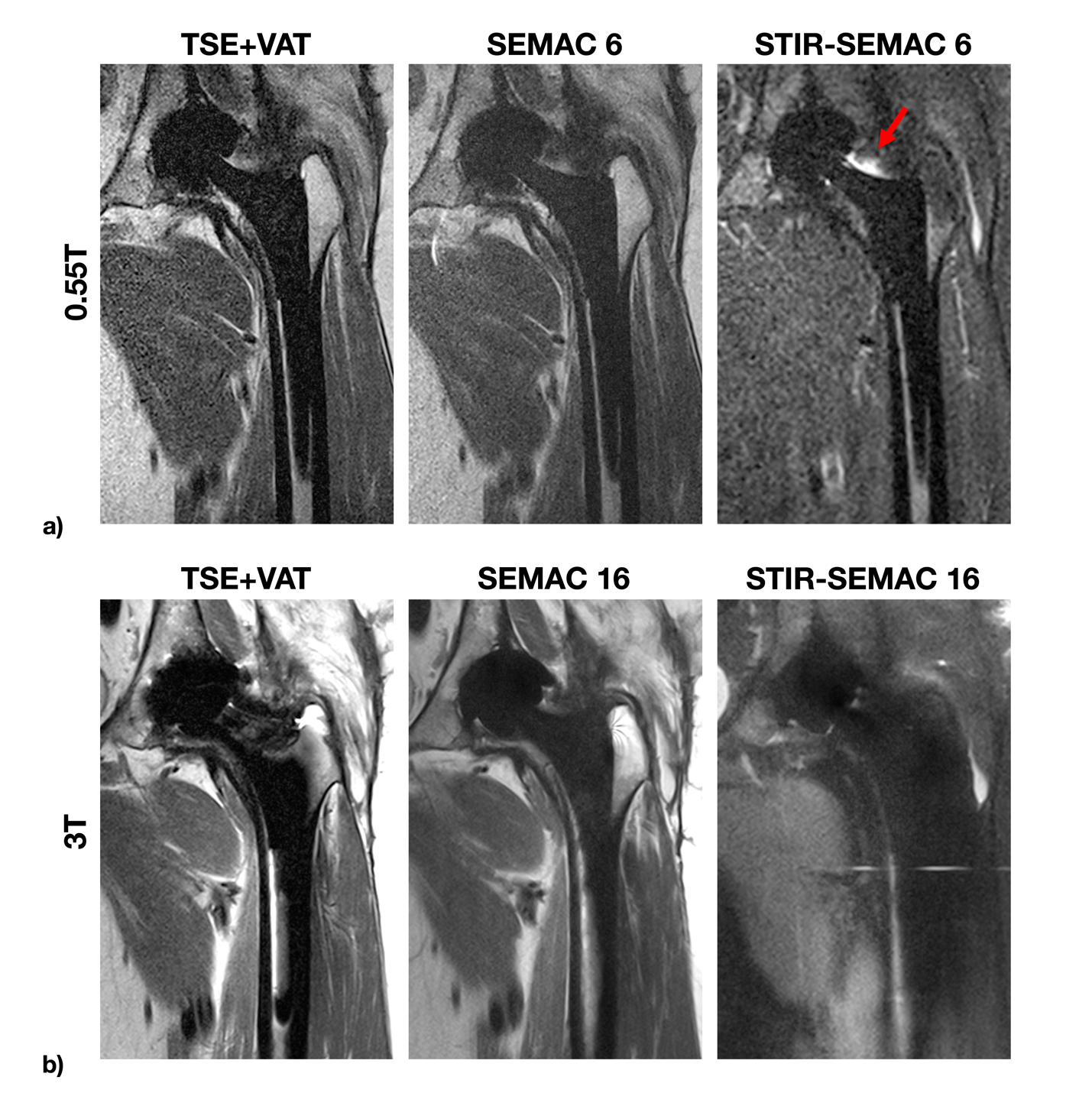
**

**Supporting Figure S3**: Comparison of TSE with VAT, SEMAC, and STIR-SEMAC images at a) 0.55T and b) 3T for a 53-year-old female with a BMI of 37.1 kg/m^2^. The fluid collection above the implant neck (red arrow) can be seen at 0.55T, while it is obscured at 3T.
